# Relationships between psoriatic arthritis composite measures of disease activity with patient-reported outcomes in phase 3 studies of tofacitinib

**DOI:** 10.1186/s13075-021-02474-2

**Published:** 2021-03-26

**Authors:** Laura C. Coates, Andrew G. Bushmakin, Oliver FitzGerald, Dafna D. Gladman, Lara Fallon, Joseph C. Cappelleri, Ming-Ann Hsu, Philip S. Helliwell

**Affiliations:** 1 University of Oxford, Oxford, UK; 2 Pfizer Inc, Groton, CT USA; 3 Conway Institute for Biomolecular Research, University College Dublin, Dublin, Ireland; 4 University of Toronto, Toronto, ON Canada; 5 Pfizer Inc, Kirkland, QC Canada; 6 Leeds Institute of Rheumatic and Musculoskeletal Medicine, University of Leeds, 2nd Floor Chapel Allerton Hospital, Chapeltown Road, Leeds, LS7 4SA UK

**Keywords:** Psoriatic arthritis, Patient-reported outcomes, Minimal disease activity

## Abstract

**Background:**

In psoriatic arthritis (PsA), further understanding of the relationships between clinical measures and patient-reported outcomes (PROs) is needed. This post hoc analysis evaluated associations between minimal disease activity (MDA) as a continuous outcome (termed ScoreMDA) or Psoriatic Arthritis Disease Activity Score (PASDAS) with selected PROs not included in the composite measures.

**Methods:**

Data from two phase 3 studies of tofacitinib in PsA (OPAL Broaden [NCT01877668; *N* = 422]; OPAL Beyond [NCT01882439; *N* = 394]) were included. MDA (binary outcome) was defined as meeting ≥5/7 criteria. For ScoreMDA, each criterion was assigned a value (1 = true; 0 = false; score range, 0–7; scores ≥5 indicated MDA). For PASDAS (score range, 0–10), higher scores indicated worse disease activity. PROs analyzed included Functional Assessment of Chronic Illness Therapy-Fatigue (FACIT-F), Patient’s Assessment of Arthritis Pain visual analog scale (Pain VAS), and EuroQoL-Five Dimensions-Three Level Health Questionnaire visual analog scale (EQ-5D-3L VAS) and utility index. Relationships were evaluated using repeated measures regression models.

**Results:**

Similar, approximately linear relationships were confirmed between PASDAS or ScoreMDA and PROs in both studies. In OPAL Broaden and OPAL Beyond, a one-point difference in PASDAS was associated with clinically relevant differences in PROs, including EQ-5D-3L VAS (− 6.7 mm, − 6.9 mm), Pain VAS (9.9 mm, 10.7 mm), and FACIT-F (− 2.8, − 3.3). A one-point difference in ScoreMDA was associated with clinically relevant differences in PROs, including EQ-5D-3L VAS (5.0 mm, 5.5 mm) and FACIT-F (1.9, 2.7) in OPAL Broaden and OPAL Beyond, respectively.

**Conclusions:**

Linear associations between PASDAS or ScoreMDA and PROs provide interpretable and quantifiable metrics between composite clinical measures and PROs, highlighting the importance of these measures in understanding the relevance of treat-to-target goals in PsA.

**Trial registration:**

ClinicalTrials.gov, NCT01877668. Registered on June 12, 2013. ClinicalTrials.gov, NCT01882439. Registered on June 18, 2013

## Background

Psoriatic arthritis (PsA) is a chronic inflammatory disease [1, 2] that occurs in approximately one-third of patients with psoriasis [3]. PsA most commonly manifests as peripheral joint disease but can also include skin and nail involvement, axial disease, enthesitis, and dactylitis [2, 3]. Consequently, patients with PsA may have considerable physical impairments [4], functional disability [4], and reduced health-related quality of life [5].

In order to effectively determine improvements in disease activity, the heterogeneous nature of PsA requires that multiple domains of the disease are evaluated following treatment [6]. This can be achieved using different clinical endpoints or composite measures that assess multiple outcomes in a single instrument [6]. Examples of composite measures commonly used in clinical studies in patients with PsA are minimal disease activity (MDA), which incorporates all clinically important aspects of PsA and includes the concepts of clinical remission and low disease activity [7, 8], and the Psoriatic Arthritis Disease Activity Score (PASDAS), a disease activity index for PsA, developed to assess changes in clinical outcomes in response to treatment [9].

Furthermore, patient-reported outcomes (PROs) that assess health-related quality of life, and symptoms such as pain and fatigue, are important components of disease assessment in the management of PsA [10]. PROs commonly used in PsA include the generic Short Form-36 Health Survey Version 2 (SF-36v2) [11], Patient’s Global Joint and Skin Assessment (PGJS) [12], Health Assessment Questionnaire-Disability Index (HAQ-DI) [13], Functional Assessment of Chronic Illness Therapy-Fatigue (FACIT-F) [14], and the EuroQoL-Five Dimensions-Three Level Health Questionnaire (EQ-5D-3L) [15]. However, the relationship between clinical measures and PROs is not well understood.

Tofacitinib is an oral Janus kinase inhibitor for the treatment of PsA. The efficacy and safety of tofacitinib 5 and 10 mg twice daily (BID) have been demonstrated in two phase 3, placebo-controlled trials [16, 17], and have been investigated in a long-term extension study [18]. In both phase 3 trials of tofacitinib in PsA, improvement in PASDAS and achievement of MDA were observed [6, 16, 17]. Moreover, improvements in several PROs with tofacitinib versus placebo were demonstrated in these studies [19, 20].

The objective of this post hoc analysis was to apply additional analytic methods to further evaluate, and thus better understand, associations of PASDAS and MDA with a set of PROs, using data from the phase 3 studies of tofacitinib in patients with active PsA.

## Methods

### Study design and patients

OPAL Broaden (NCT01877668) [16] and OPAL Beyond (NCT01882439) [17] were phase 3, multicenter, placebo-controlled, double-blind, randomized studies of tofacitinib in patients with active PsA. OPAL Broaden was a 12-month study conducted from January 2014 to December 2015 [16], and OPAL Beyond was a 6-month study conducted from June 2013 to April 2016 [17]. Both studies were conducted in accordance with the Declaration of Helsinki and the International Conference on Harmonisation Guidelines for Good Clinical Practice. All patients provided informed consent.

Full eligibility criteria for both studies have been previously published [16, 17]. In both studies, patients (aged ≥18 years) had a diagnosis (≥6 months previously) of active PsA according to ClASsification criteria for Psoriatic ARthritis (CASPAR) [21] and, at screening, had ≥3 tender/painful and 3 swollen joints, and active plaque psoriasis. In OPAL Broaden, patients had an inadequate response to ≥1 conventional synthetic disease-modifying antirheumatic drug (csDMARD) and were tumor necrosis factor inhibitor (TNFi)-naive. In OPAL Beyond, patients had an inadequate response to ≥1 TNFi.

Patients were randomized to tofacitinib 5 mg BID, tofacitinib 10 mg BID, placebo switching to tofacitinib 5 mg BID at month 3, or placebo switching to tofacitinib 10 mg BID at month 3 [16, 17]. In OPAL Broaden only, patients were randomized to adalimumab 40 mg subcutaneous injection once every 2 weeks (Q2W) [16]. All patients received a stable dose of a single csDMARD [16, 17].

### Assessments

PASDAS and MDA were assessed at baseline and at months 1, 3, 6, 9, and 12 in OPAL Broaden, and at baseline and at months 1, 3, and 6 in OPAL Beyond.

PASDAS (score range, 0–10) is composed of the following measures: Patient Global Assessment of PsA visual analog scale (VAS); Physician Global Assessment of PsA VAS; tender and swollen joint counts; Leeds Enthesitis Index; tender dactylitis count; SF-36v2 Physical Component Summary (PCS) score; and C-reactive protein measurement, with each component weighted before contributing to the final score [9, 22]. Very low, low, and high disease activity were defined as PASDAS cutoffs of 1.9, 3.2, and 5.4, respectively [23, 24].

MDA and very low disease activity in PsA are defined as meeting ≥5 and 7, respectively, of the 7 following criteria: tender joint count ≤1; swollen joint count ≤1; Psoriasis Activity and Severity Index (PASI) ≤1 or body surface area ≤3%; patient global disease activity VAS ≤20; Patient’s Assessment of Arthritis Pain VAS (Pain VAS) ≤15; HAQ-DI score ≤0.5; and tender entheseal points (using Leeds Enthesitis Index) ≤1 [7, 24].

The PROs included in this analysis were EQ-5D-3L VAS and utility index (UI); FACIT-F; PGJS-VAS; PGJS-VAS-Psoriasis question (PGJS-VAS-PsO); SF-36v2, acute; Pain VAS; and HAQ-DI (Additional Table 1). PROs included within each composite clinical measure (e.g., SF-36v2 PCS in PASDAS and HAQ-DI in MDA) were not assessed with that measure. PROs were analyzed up to month 12 in OPAL Broaden and month 6 in OPAL Beyond. The criteria for clinically important differences (CIDs), defined as the differences between two treatment groups that can be considered clinically relevant, are presented in Additional Table 1.

### Statistical analysis

This post hoc analysis included all available data from OPAL Broaden and OPAL Beyond. Data from all treatment groups were pooled for this analysis, with no imputation for missing values. Any PRO included in the composite measure was not included in the analysis of associations between that PRO and the composite measure.

The scoring of MDA was augmented (hereafter referred to as ScoreMDA) by considering each criterion as an individual item and assigning each criterion a value of 1 if true and 0 if false, to give a total score range of 0–7. Total scores ≥5 indicated the patient had MDA. As a result, ScoreMDA was a measurement scale represented by a continuous outcome with exact backward compatibility with the binary MDA, and could be used to enhance the interpretation of the composite, binary MDA outcome via relationships with PROs.

A repeated measures longitudinal regression model was used to investigate the relationship between PASDAS or ScoreMDA and each PRO [25, 26]. PASDAS or ScoreMDA was used as a continuous anchor, meaning that a linear relationship was imposed between each PRO and the composite measure. A sensitivity analysis was performed to assess the linearity assumption using PASDAS or ScoreMDA as a categorical anchor (represented by integer values 0–10 or 0–7, respectively). Using PASDAS or ScoreMDA as a categorical anchor does not impose any functional relationship between anchor and outcome.

## Results

This analysis used data from 422 patients from OPAL Broaden (*N* = 105, placebo; *N* = 107, tofacitinib 5 mg BID; *N* = 104, tofacitinib 10 mg BID; *N* = 106, adalimumab 40 mg Q2W) and 394 patients from OPAL Beyond (*N* = 131, placebo; *N* = 131, tofacitinib 5 mg BID; *N* = 132, tofacitinib 10 mg BID). Demographics and baseline disease characteristics for these patients have been reported previously and were generally balanced across groups [16, 17]. Mean PASDAS and baseline PRO scores were generally comparable across treatment groups [6, 19, 20].

### Relationships between PASDAS or ScoreMDA and PROs

**Fig. 1:**
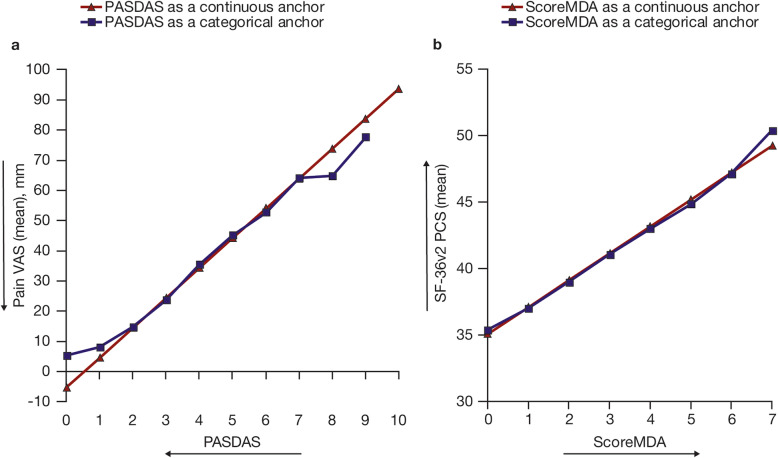
Estimated relationship between PASDAS/Pain VAS (**a**) and ScoreMDA/SF-36v2 PCS (**b**). Data from OPAL Broaden (*N* = 422). SF-36v2 PCS: norm-based scores were used (a score of 50 representing the mean for the general population, with higher scores indicating less impairment); ScoreMDA: continuous MDA with values from 0 to 7 (0–4, no MDA; 5–7, MDA). Arrows indicate the direction of improvement. MDA, minimal disease activity; Pain VAS, Patient’s Assessment of Arthritis Pain visual analog scale; PASDAS, Psoriatic Arthritis Disease Activity Score; PCS, Physical Component Summary; SF-36v2, Short Form-36 Health Survey Version 2, acute

Relationships between PASDAS and PROs, and also between ScoreMDA and PROs, were very close when using PASDAS or ScoreMDA as a continuous anchor, compared with a categorical anchor. Figure 1 shows an example of the linear relationship between PASDAS and Pain VAS, and ScoreMDA and SF-36v2 PCS scores in OPAL Broaden. The close relationship was consistent across all PROs evaluated in both OPAL Broaden and OPAL Beyond (data not shown), and confirms the linearity assumption.

**Fig. 2:**
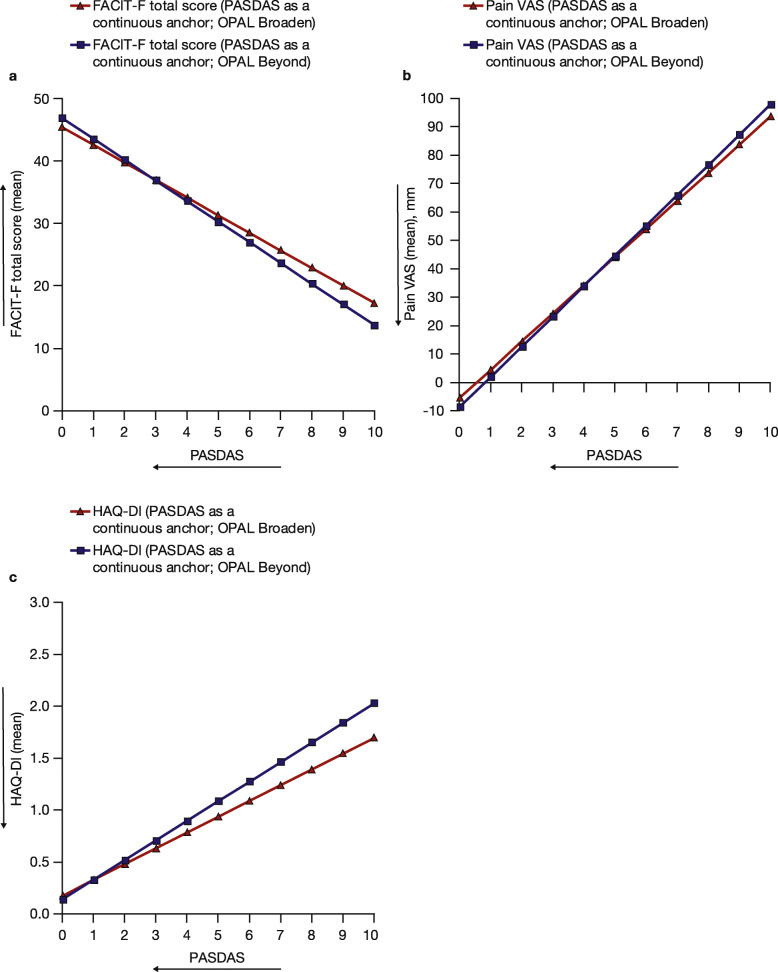
Estimated relationships between PASDAS and FACIT-F total score (**a**), Pain VAS (**b**), and HAQ-DI (**c**). Data from OPAL Broaden (*N* = 422) and OPAL Beyond (*N* = 394); all treatment groups from each phase 3 study were pooled for analysis. Arrows indicate the direction of improvement. FACIT-F, Functional Assessment of Chronic Illness Therapy-Fatigue; HAQ-DI, Health Assessment Questionnaire-Disability Index; Pain VAS, Patient’s Assessment of Arthritis Pain visual analog scale; PASDAS, Psoriatic Arthritis Disease Activity Score; VAS visual analog scale

**Fig. 3:**
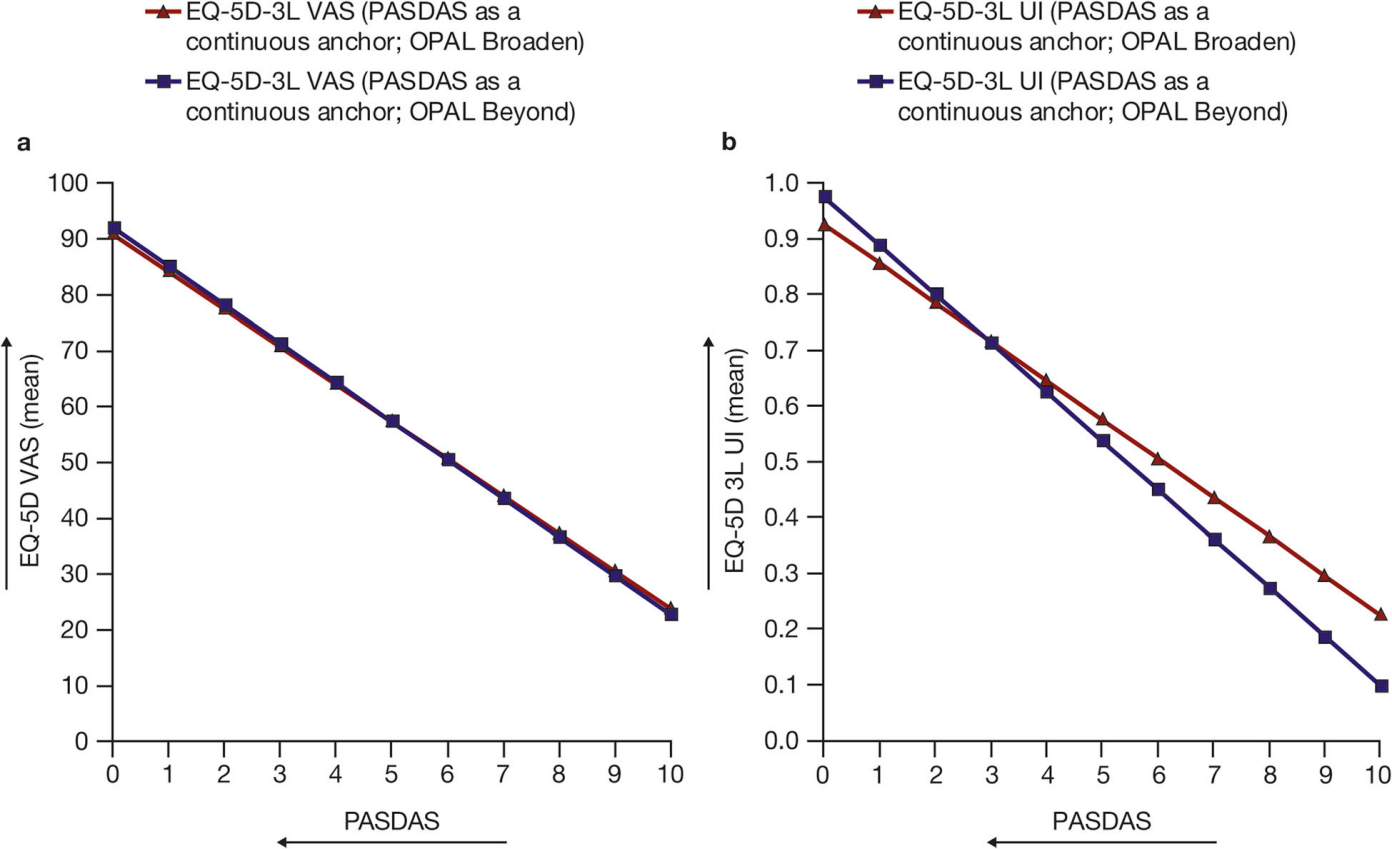
Estimated relationships between PASDAS and EQ-5D-3L VAS (**a**) and EQ-5D-3L UI (**b**). Data from OPAL Broaden (*N* = 422) and OPAL Beyond (*N* = 394); all treatment groups from each phase 3 study were pooled for analysis. Arrows indicate the direction of improvement. EQ-5D-3L, EuroQoL-Five Dimensions-Three Level Health Questionnaire; PASDAS, Psoriatic Arthritis Disease Activity Score; UI, utility index; VAS, visual analog scale

The estimated relationships between PASDAS as a continuous anchor and FACIT-F total score, Pain VAS, and HAQ-DI (Fig. 2), and EQ-5D-3L (Fig. 3), were similar in both OPAL Broaden and OPAL Beyond. For all other PROs tested, the estimated relationships between PASDAS and each PRO measure were also similar (Additional Fig. 1).

**Fig. 4:**
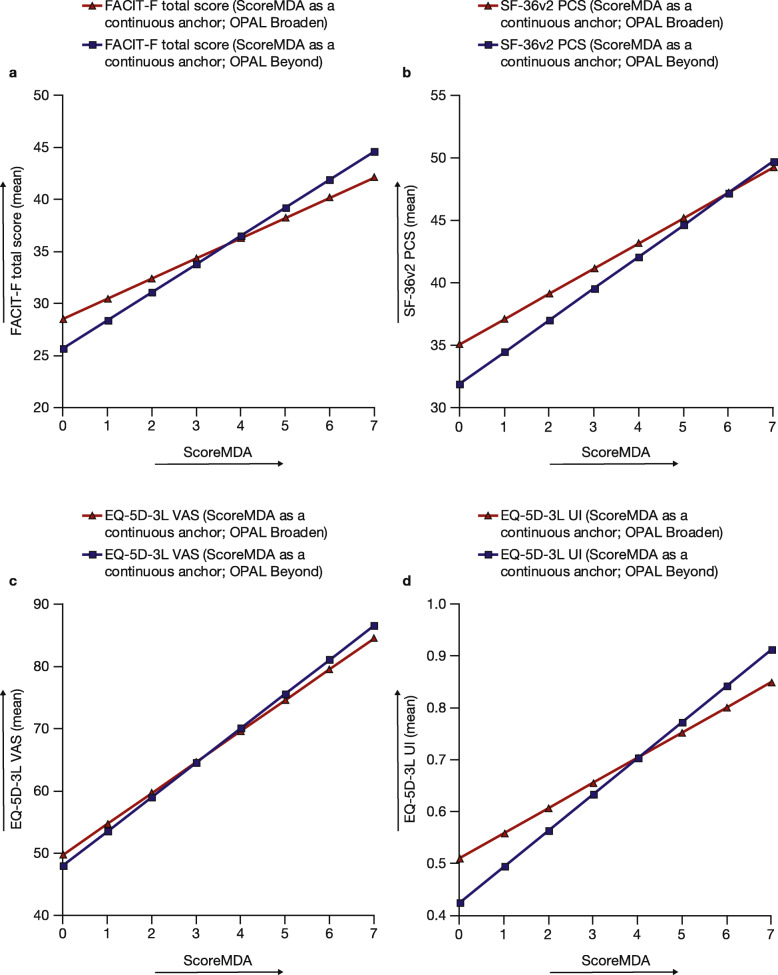
Estimated relationships between ScoreMDA and FACIT-F total score (**a**), SF-36v2 PCS (**b**), EQ-5D-3L VAS (**c**), and EQ-5D-3L UI (**d**). Data from OPAL Broaden (*N* = 422) and OPAL Beyond (*N* = 394); all treatment groups from each phase 3 study were pooled for analysis. SF-36v2 PCS: norm-based scores were used (a score of 50 representing the mean for the general population, with higher scores indicating less impairment); ScoreMDA: continuous MDA with values from 0 to 7 (0–4, no MDA; 5–7, MDA). EQ-5D-3L, EuroQoL-Five Dimensions-Three Level Health Questionnaire; FACIT-F, Functional Assessment of Chronic Illness Therapy-Fatigue; MDA, minimal disease activity; PCS, Physical Component Summary; SF-36v2, Short Form-36 Health Survey Version 2, acute; UI, utility index, VAS, visual analog scale

When ScoreMDA was used as a continuous anchor, the estimated relationships between ScoreMDA and FACIT-F total score, SF-36v2 PCS, and EQ-5D-3L were similar in both OPAL Broaden and OPAL Beyond (Fig. 4). Data for other PROs are shown in Additional Fig. 2 and demonstrate similar results in both OPAL Broaden and OPAL Beyond for each PRO.

### Relationship between a one-point difference in PASDAS or ScoreMDA and PROs

The mean differences in PRO measures associated with a one-point difference in PASDAS in OPAL Broaden (where patients had an inadequate response to ≥1 csDMARD and were TNFi-naive) and in OPAL Beyond (where patients had an inadequate response to ≥1 TNFi) are shown in Table 1. Differences in EQ-5D-3L VAS and EQ-5D-3L UI met the criteria for CID (Additional Table 1), and differences for FACIT-F total score, FACIT-F experience domain (ED), and FACIT-F impact domain (ID) all approached clinical importance based on the CID criteria.

**Table 1:** Mean score difference in PROs associated with a one-point difference in PASDAS or ScoreMDA

PRO*	Mean score difference (95% CI) in PROs associated with a one-point difference	
	PASDAS	ScoreMDA
EQ-5D-3L VAS, mm		
OPAL Broaden	−6.7 (− 7.2, − 6.3)	5.0 (4.6, 5.4)
OPAL Beyond	− 6.9 (− 7.5, − 6.4)	5.5 (4.9, 6.1)
EQ-5D-3L UI		
OPAL Broaden	− 0.1 (− 0.1, − 0.1)	0.0 (0.0, 0.1)
OPAL Beyond	−0.1 (− 0.1, − 0.1)	0.1 (0.1, 0.1)
FACIT-F total score		
OPAL Broaden	−2.8 (− 3.0, − 2.6)	1.9 (1.8, 2.1)
OPAL Beyond	−3.3 (− 3.6, − 3.1)	2.7 (2.5, 3.0)
FACIT-F ED		
OPAL Broaden	−1.3 (− 1.4, − 1.2)	0.9 (0.8, 0.9)
OPAL Beyond	−1.4 (− 1.6, − 1.3)	1.2 (1.1, 1.3)
FACIT-F ID		
OPAL Broaden	−1.6 (− 1.7, − 1.4)	1.1 (1.0, 1.2)
OPAL Beyond	−1.9 (− 2.0, − 1.7)	1.5 (1.4, 1.7)
PGJS-VAS, mm		
OPAL Broaden	12.0 (11.6, 12.4)	−7.6 (− 8.0, − 7.2)
OPAL Beyond	12.6 (12.1, 13.1)	− 9.2 (− 9.9, − 8.6)
PGJS-VAS-PsO, mm		
OPAL Broaden	9.9 (9.4, 10.5)	−6.0 (− 6.5, − 5.6)
OPAL Beyond	10.4 (9.7, 11.1)	− 7.2 (− 8.0, − 6.5)
SF-36v2 PCS		
OPAL Broaden	NA	2.0 (1.9, 2.2)
OPAL Beyond	NA	2.5 (2.3, 2.7)
SF-36v2 PF		
OPAL Broaden	NA	2.1 (2.0, 2.3)
OPAL Beyond	NA	2.3 (2.1, 2.5)
Pain VAS, mm		
OPAL Broaden	9.9 (9.4, 10.4)	NA
OPAL Beyond	10.7 (10.1, 11.2)	NA
HAQ-DI		
OPAL Broaden	0.2 (0.1, 0.2)	NA
OPAL Beyond	0.2 (0.2, 0.2)	NA

SF-36v2 PCS: norm-based scores were used (a score of 50 representing the mean for the general population, with higher scores indicating less impairment); ScoreMDA: continuous MDA with values from 0 to 7 (0–4, no MDA; 5–7, MDA)

*CI* confidence interval, *ED* experience domain, *EQ-5D-3L* EuroQoL-Five Dimensions-Three Level Health Questionnaire, *FACIT* Functional Assessment of Chronic Illness Therapy-Fatigue, *HAQ-DI* Health Assessment Questionnaire-Disability Index, *ID* impact domain, *MDA* minimal disease activity, *NA* not assessed, *Pain VAS* Patient’s Assessment of Arthritis Pain visual analog scale, *PASDAS* Psoriatic Arthritis Disease Activity Score, *PCS* Physical Component Summary, *PF* physical functioning, *PGJS-VAS* Patient’s Global Joint and Skin Assessment visual analog scale, *PGJS-VAS-PsO* PGJS-VAS psoriasis question, *PRO* patient-reported outcome, *SF-36v2* Short Form-36 Health Survey Version 2, acute, *VAS* visual analog scale

*OPAL Broaden, *N* = 422; OPAL Beyond, *N* = 394; all treatment groups from each phase 3 study were pooled for analysis

Table 1 presents the mean differences in PRO scores associated with a one-point difference in ScoreMDA. For EQ-5D-3L VAS, a one-point difference in ScoreMDA was associated with clinically relevant differences as defined by CID criteria. For EQ-5D-3L UI, FACIT-F total score, FACIT-F ED, and FACIT-F ID, mean differences associated with a one-point difference in ScoreMDA approached clinical importance.

### Relationship between PASDAS and ScoreMDA cutoffs for disease activity and PROs

Mean scores corresponding to PASDAS cutoffs for very low, low, and high disease activity mean scores (1.9, 3.2, and 5.4, respectively) are shown in Table 2. For each PRO, scores for each disease activity cutoff were similar in OPAL Broaden and OPAL Beyond. PRO scores corresponding to MDA (ScoreMDA cutoff of 5 points) were also similar for each PRO in OPAL Broaden and OPAL Beyond (Table 2).

**Table 2:** Mean PRO scores corresponding to PASDAS and ScoreMDA cutoffs for disease activity

PRO*	Mean scores (95% CI) corresponding to PASDAS cutoffs for disease activity			Mean scores (95% CI) corresponding to MDA
	Very low: 1.9	Low: 3.2	High: 5.4	5 points
EQ-5D-3L VAS				
OPAL Broaden	78.2 (76.6, 79.8)	69.5 (68.2, 70.7)	54.7 (53.4, 56.0)	74.6 (73.0, 76.2)
OPAL Beyond	78.9 (76.7, 81.1)	69.9 (68.2, 71.6)	54.7 (53.2, 56.1)	75.5 (73.2, 77.9)
EQ-5D-3L UI				
OPAL Broaden	0.8 (0.8, 0.8)	0.7 (0.7, 0.7)	0.5 (0.5, 0.6)	0.8 (0.7, 0.8)
OPAL Beyond	0.8 (0.8, 0.8)	0.7 (0.7, 0.7)	0.5 (0.5, 0.5)	0.8 (0.7, 0.8)
FACIT-F total score				
OPAL Broaden	40.1 (39.2, 41.0)	36.4 (35.6, 37.2)	30.2 (29.4, 31.0)	38.2 (37.3, 39.1)
OPAL Beyond	40.6 (39.5, 41.7)	36.3 (35.3, 37.2)	29.0 (28.1, 29.8)	39.2 (38.0, 40.4)
FACIT-F ED				
OPAL Broaden	14.3 (14.0, 14.7)	12.7 (12.3, 13.0)	9.9 (9.6, 10.2)	13.4 (13.0, 13.8)
OPAL Beyond	14.4 (13.9, 15.0)	12.6 (12.2, 13.0)	9.4 (9.0, 9.8)	13.9 (13.4, 14.4)
FACIT-F ID				
OPAL Broaden	25.8 (25.2, 26.3)	23.7 (23.2, 24.2)	20.3 (19.8, 20.8)	24.9 (24.3, 25.4)
OPAL Beyond	26.2 (25.5, 26.9)	23.7 (23.1, 24.3)	19.6 (19.0, 20.1)	25.4 (24.7, 26.1)
PGJS-VAS, mm				
OPAL Broaden	12.6 (11.1, 14.0)	28.2 (27.1, 29.3)	54.7 (53.6, 55.8)	22.1 (20.4, 23.7)
OPAL Beyond	11.4 (9.5, 13.3)	27.8 (26.3, 29.2)	55.4 (54.1, 56.7)	20.2 (17.7, 22.7)
PGJS-VAS-PsO, mm				
OPAL Broaden	11.0 (9.1, 12.9)	24.0 (22.4, 25.5)	45.9 (44.2, 47.5)	18.7 (16.7, 20.6)
OPAL Beyond	8.6 (6.0, 11.2)	22.2 (20.2, 24.2)	45.2 (43.3, 47.0)	16.5 (13.6, 19.5)
SF-36v2 PCS				
OPAL Broaden	NA	NA	NA	45.2 (44.5, 45.8)
OPAL Beyond	NA	NA	NA	44.6 (43.7, 45.5)
SF-36v2 PF				
OPAL Broaden	NA	NA	NA	45.3 (44.5, 46.1)
OPAL Beyond	NA	NA	NA	43.4 (42.3, 44.5)
Pain VAS				
OPAL Broaden	13.4 (11.8, 15.0)	26.3 (25.0, 27.5)	48.0 (46.7, 49.4)	NA
OPAL Beyond	11.5 (9.3, 13.8)	25.4 (23.7, 27.1)	48.8 (47.4, 50.3)	NA
HAQ-DI				
OPAL Broaden	0.5 (0.4, 0.5)	0.7 (0.6, 0.7)	1.0 (1.0, 1.0)	NA
OPAL Beyond	0.5 (0.4, 0.6)	0.7 (0.7, 0.8)	1.2 (1.1, 1.2)	NA

SF-36v2 PCS: norm-based scores were used (a score of 50 representing the mean for the general population, with higher scores indicating less impairment); ScoreMDA: continuous MDA with values from 0 to 7 (0–4, no MDA; 5–7, MDA)

*CI* confidence interval, *ED* experience domain, *EQ-5D-3L* EuroQoL-Five Dimensions-Three Level Health Questionnaire, *FACIT-F* Functional Assessment of Chronic Illness Therapy-Fatigue, *HAQ-DI* Health Assessment Questionnaire-Disability Index, *ID* impact domain, *MDA* minimal disease activity, *NA* not assessed, *Pain VAS* Patient’s Assessment of Arthritis Pain visual analog scale, *PASDAS* Psoriatic Arthritis Disease Activity Score, *PCS* Physical Component Summary, *PF* physical functioning, *PGJS-VAS* Patient’s Global Joint and Skin Assessment visual analog scale, *PGJS-VAS-PsO* PGJS-VAS psoriasis question, *PRO* patient-reported outcome, *SF-36v2* Short Form-36 Health Survey Version 2, acute, *VAS* visual analog scale

*OPAL Broaden, *N* = 422; OPAL Beyond, *N* = 394; all treatment groups from each phase 3 study were pooled for analysis

## Discussion

This post hoc analysis investigated the relationship between composite clinical measures (PASDAS and ScoreMDA) and a set of PROs (those not included within each of the clinical measures) using data from two phase 3 studies of tofacitinib (OPAL Broaden and OPAL Beyond) in patients with active PsA.

Achievement of MDA is normally evaluated as a binary outcome measure, with MDA generally defined as patients having to achieve at least 5 of 7 criteria relating to improvements in tender and swollen joint counts, pain, enthesitis, PASI, Patient Global Assessment of Disease Activity, and HAQ-DI scores [7]. Here, a continuous MDA outcome, ScoreMDA, was proposed as an alternative to the binary MDA measure to facilitate the analysis. Using this approach, each component of the MDA criteria was considered as an individual item to give a score range of 0–7. A score ≥5 indicated that the patient had MDA, consistent with the binary outcome measure; however, by augmenting MDA, the continuous measure allowed greater interpretation of the relationships with PROs.

Results of the analysis presented here demonstrated markedly close relationships between both PASDAS and ScoreMDA and the PRO measures, irrespective of whether PASDAS or ScoreMDA was used as a categorical or continuous anchor. In both studies, an approximately linear relationship was confirmed between PASDAS and selected PROs, and a close linear relationship between ScoreMDA and selected PROs. Of particular note, our analysis provided further insights into the relationship between PASDAS and ScoreMDA and fatigue, a core domain of PsA [27]. Fatigue is under-represented in composite scores such as PASDAS and MDA, which is attributed to it not necessarily being related to disease activity.

In general, PRO changes with ScoreMDA and PASDAS were higher in OPAL Beyond than in OPAL Broaden, which may be attributed to the different patient populations. Patients in OPAL Broaden had an inadequate response to ≥1 csDMARD and were TNFi-naive, whereas patients in OPAL Beyond had an inadequate response to ≥1 TNFi. Patients in OPAL Beyond had a longer disease duration [16, 17], and potentially less reversible changes in their impact measures.

Furthermore, the results suggest that achieving low disease activity (as measured by PASDAS) or MDA (as measured by ScoreMDA) translates into clinically meaningful improvements in various PROs. This finding is consistent with the results of previous analyses using PASDAS [28] or MDA (as a binary outcome) [29]. A one-point difference on PASDAS and ScoreMDA was associated with differences in PROs approaching CIDs. Moreover, while these results indicate that improvements in any one MDA criterion or a PASDAS score change of one point were associated with an improvement in PRO scores approaching clinical relevance, improvements in any two criteria of MDA, or two points for PASDAS, were associated with an improvement in PRO scores that exceeded the CID.

A close association was observed between EQ-5D-3L VAS and EQ-5D-3L UI, and both PASDAS and ScoreMDA, and was consistent in both OPAL Broaden and OPAL Beyond. As health-related quality of life can be estimated from EQ-5D utility scores, calculated from their mapped relationship with MDA or PASDAS scores, these findings may facilitate the calculation of quality-adjusted life-years and inform further economic evaluations of healthcare interventions [30, 31].

The content validity of the PASDAS composite measure has been questioned based on the lack of a component assessing pain [32], which is a core domain of PsA [27]. A recent study evaluated the relationship between PASDAS and the composite Disease Activity Index for Psoriatic Arthritis (DAPSA) and the threshold for Patient Acceptable Symptom State (PASS) [33]. For PASDAS, the PASS threshold was within the moderate disease activity range, whereas for DAPSA, this was within the low disease activity range. The authors speculated that this difference may be due to the absence of a pain measure in PASDAS [33]. Our findings demonstrated a linear relationship between PASDAS and Pain VAS, regardless of whether PASDAS was used as a continuous or categorical anchor, confirming the utility of PASDAS.

The psychometric association between PROs and other clinical measures has been reported previously [33–35]; however, the association between PROs and composite clinical measures in PsA using the methodology employed in this analysis has, to our knowledge, not been evaluated. The results of this analysis, therefore, provide further insights into, and contextualization of, the relationship between clinical measures and PROs beyond those already described.

An analysis of data from the phase 3 FUTURE 2 trial (NCT01752634) of the human immunoglobulin G1 monoclonal antibody secukinumab in patients with active PsA demonstrated that patients achieving PASDAS-defined remission or low disease activity had greater improvements in PROs (health-related quality of life, fatigue, and physical functioning) than in patients with high disease activity [28]. Similarly, results of the ADEPT trial (NCT00646386) of adalimumab in patients with active PsA demonstrated that achievement of MDA was associated with clinically significant improvements in PROs [36]. Improved health-related quality of life outcomes (using the PsA Impact of Disease [PsAID] questionnaire) were also reported for patients achieving MDA, versus those who did not, in a cross-sectional study of csDMARDs or biologic DMARDs in patients with PsA [37]. The findings of this analysis are consistent with the results of an analysis of pooled data from two studies of ixekizumab (NCT01695239) that evaluated the relationship between disease activity and quality of life and productivity. Greater improvements in SF-36v2 PCS, SF-36v2 physical functioning, EQ-5D-5 Level VAS, and EQ-5D health state index were observed in patients who achieved MDA compared with those who did not [34].

A potential limitation of this analysis was that data were used from just two clinical trials; however, these trials included different patient populations, and the similar results shown across OPAL Broaden and OPAL Beyond provides validation of this approach. Consistent with high-quality scientific research, it would also be prudent to conduct additional research using larger, real-world datasets.

## Conclusions

In conclusion, the findings of this post hoc analysis from two phase 3 studies of tofacitinib in patients with active PsA suggest that an approximately linear relationship exists between PASDAS and selected PROs, and a close linear relationship exists between ScoreMDA and selected PROs. These findings provide more detailed insights into the relationships between clinical measures and PROs in PsA, which could aid their interpretation and enhance the assessment of disease activity. In addition, these results support the treat-to-target strategy and highlight the importance of the composite clinical measures in understanding the relevance of treat-to-target goals in PsA.

## Supplementary Information

**Additional file 1 **: **Table S1.** PROs included in the analysis. Table listing PROs and their descriptions included in the analysis of ScoreMDA and PASDAS.

**Additional file 2 **: **Fig. S1.** Estimated relationships between PASDAS and PGJS-VAS (a), PGJS-VAS-PsO (b), FACIT-F ED (c), and FACIT-F ID (d). Figure showing the estimated relationships between PASDAS as a continuous anchor and PGJS-VAS, PGJS-VAS-PsO, FACIT-F ED, and FACIT-F ID in OPAL Broaden and OPAL Beyond.

**Additional file 3 **: **Fig. S2.** Estimated relationships between ScoreMDA and PGJS-VAS (a), PGJS-VAS-PsO (b), FACIT-F ED (c), and FACIT-F ID (d). Figure showing the estimated relationships between ScoreMDA as a continuous anchor and PGJS-VAS, PGJS-VAS-PsO, FACIT-F ED, and FACIT-F ID in OPAL Broaden and OPAL Beyond.

## Data Availability

Upon request, and subject to certain criteria, conditions, and exceptions (see https://www.pfizer.com/science/clinical-trials/trial-data-and-results for more information), Pfizer will provide access to individual de-identified participant data from Pfizer-sponsored global interventional clinical studies conducted for medicines, vaccines, and medical devices (1) for indications that have been approved in the USA and/or EU or (2) in programs that have been terminated (i.e., development for all indications has been discontinued). Pfizer will also consider requests for the protocol, data dictionary, and statistical analysis plan. Data may be requested from Pfizer trials 24 months after study completion. The de-identified participant data will be made available to researchers whose proposals meet the research criteria and other conditions, and for which an exception does not apply, via a secure portal. To gain access, data requestors must enter into a data access agreement with Pfizer.

## Abbreviations

BID: Twice daily
CASPAR: ClASsification criteria for Psoriatic Arthritis
CI: Confidence interval
CID: Clinically important difference
csDMARD: Conventional synthetic disease-modifying antirheumatic drug
DAPSA: Disease Activity Index for Psoriatic Arthritis
ED: Experience domain
EQ-5D-3L: EuroQoL-Five Dimensions-Three Level Health Questionnaire
FACIT-F: Functional Assessment of Chronic Illness Therapy-Fatigue
HAQ-DI: Health Assessment Questionnaire-Disability Index
ID: Impact domain
MDA: Minimal disease activity
NA: Not assessed
Pain VAS: Patient’s Assessment of Arthritis Pain visual analog scale
PASDAS: Psoriatic Arthritis Disease Activity Score
PASI: Psoriasis Activity and Severity Index
PASS: Patient Acceptable Symptom State
PCS: Physical Component Summary
PF: Physical functioning
PGJS: Patient’s Global Joint and Skin Assessment
PGJS-VAS-PsO: Patient’s Global Joint and Skin Assessment-visual analog scale-psoriasis question
PRO: Patient-reported outcome
PsA: Psoriatic arthritis
PsAID: PsA Impact of Disease
Q2W: Every 2 weeks
ScoreMDA: Minimal disease activity as a continuous outcome
SF-36v2: Short Form-36 Health Survey Version 2, acute
TNFi: Tumor necrosis factor inhibitor
UI: Utility index
VAS: Visual analog scale
