# Supplementary material for: Relationships between psoriatic arthritis composite measures of disease activity with patient-reported outcomes in phase 3 studies of tofacitinib
Source: Arthritis Res Ther. 2021 Mar 26;23:94. doi: 10.1186/s13075-021-02474-2 (PMC7995583; doi:10.1186/s13075-021-02474-2)
Supplement: Supplementary file 1 — Additional file 1 : Table S1. PROs included in the analysis. Table listing PROs and their descriptions included in the analysis of ScoreMDA and PASDAS. [file 13075_2021_2474_MOESM1_ESM.docx]

**Additional material**

**Additional Table 1** PROs included in the analysis

| **PRO** | **Description** | **CID [reference]** | **Included in analysis of ScoreMDA^*^** | **Included in analysis of PASDAS^†^** |
| --- | --- | --- | --- | --- |
| EQ-5D-3L | VAS   - Total score range=0–100 (0=worst health you can imagine; 100=best health you can imagine) | 4.2–14.8 mm [1] | Yes | Yes |
|  | Health UI (UK weighted)   - Negative values=a state worse than death; 0=state equal to death; 1.0=perfect health | 0.06–0.12 points [2] | Yes | Yes |
| FACIT-F | - FACIT-F total (score range 0–52; increasing score=less fatigue) | 3.1 points [3] | Yes | Yes |
|  | - FACIT-F ED (score range 0–20; increasing score=decreased experience of fatigue) | 1.5 points [3] | Yes | Yes |
|  | - FACIT-F ID (score range 0–32; increasing score=decreased fatigue impact on patient’s life) | 1.7 points [3] | Yes | Yes |
| PGJS-VAS | - Patients answer the question ‘In all the ways in which your PSORIASIS and ARTHRITIS, as a whole, affects you, how would you rate the way you felt over the past week?’ using a 100 mm VAS (0=excellent and 100=poor) | NA | Yes | Yes |
| PGJS-VAS-PsO | - Patients answer the question ‘In all the ways your PSORIASIS affects you, how would you rate the way in which you felt over the past week?’ using a 100 mm VAS (0=excellent and 100=poor) | NA | Yes | Yes |
| SF-36v2  PCS  PF | - Transformed norm-based scores with score of 50 (SD=10) representing mean and SD for general population | 3.0 points [4, 5] | Yes | No |
| Pain VAS | - Patients assess the severity of their current arthritis pain using a 100 mm VAS (0=no pain and 100=most severe pain) | NA | No | Yes |
| HAQ-DI | - Total score range 0–3 (increasing scores indicate worse functioning [0=no functional impairment and 3=complete impairment]) | 0.35 [6] | No | Yes |
| PtGA VAS^‡^ | - NA | NA | No | No |

^*^Clinical/physician-reported measures included in the assessment of MDA and not included in this analysis are TJC, SJC, PASI, and LEI
^†^Clinical/physician-reported measures included in the assessment of PASDAS and not included in this analysis are TJC, SJC, LEI, LDI, CRP, and PGA
^‡^Included in both MDA and PASDAS composite measures, therefore, not included in this analysis
SF-36v2 PCS: norm-based scores were used (a score of 50 representing the mean for the general population, with higher scores indicating less impairment); ScoreMDA: continuous MDA with values from 0–7 (0–4, no MDA; 5–7, MDA)
*CID* clinically important difference, *CRP* C-reactive protein, *ED* experience domain, *EQ-5D-3L* EuroQoL-Five Dimensions-Three Level Health Questionnaire, *FACIT-F* Functional Assessment of Chronic Illness Therapy-Fatigue, *HAQ-DI* Health Assessment Questionnaire-Disability Index, *ID* impact domain, *LDI* Leeds Dactylitis Index, *LEI* Leeds Enthesitis Index, *NA* not applicable, *Pain VAS* Patient’s Assessment of Arthritis Pain visual analog scale, *PASDAS* Psoriatic Arthritis Disease Activity Score, *PASI* Psoriasis Area Severity Index, *PCS* Physical Component Summary, *PF* physical functioning, *PGA* Physician Global Assessment, *PGJS-VAS* Patient’s Global Joint and Skin Assessment visual analog scale, *PGJS-VAS-PsO* PGJS-VAS Psoriasis question, *PRO* patient-reported outcome, *PtGA* Patient Global Assessment of Disease Activity, *SD* standard deviation, *SF-36v2* Short Form-36 Health Survey Version 2, acute, *SJC* swollen joint count, *TJC* tender joint count, UI utility index, *VAS* visual analog scale

**References (for Additional Table 1)**

1. Coteur G, Feagan B, Keininger DL, Kosinski M. Evaluation of the meaningfulness of health-related quality of life improvements as assessed by the SF-36 and the EQ-5D VAS in patients with active Crohn's disease. Aliment Pharmacol Ther. 2009;29:1032-41.

2. Pickard AS, Neary MP, Cella D. Estimation of minimally important differences in EQ-5D utility and VAS scores in cancer. Health Qual Life Outcomes. 2007;5:70.

3. Cella D, Wilson H, Shalhoub H, Revicki DA, Cappelleri JC, Bushmakin AG, et al. Content validity and psychometric evaluation of Functional Assessment of Chronic Illness Therapy-Fatigue in patients with psoriatic arthritis. J Patient Rep Outcomes. 2019;3:30.

4. Ware JE, Kosinski Jr M, Bjorner JB, Turner-Bowker DM, Gandek B, Maruish ME. User’s Manual for the SF-36v2^®^ Health Survey. 2nd ed. Lincoln (RI): QualityMetric Incorporated; 2007.

5. Maruish ME. User's Manual for the SF-36v2^®^ Health Survey. 3rd ed. Lincoln (RI): QualityMetric Incorporated; 2011.

6. Mease PJ, Woolley JM, Bitman B, Wang BC, Globe DR, Singh A. Minimally important difference of Health Assessment Questionnaire in psoriatic arthritis: relating thresholds of improvement in functional ability to patient-rated importance and satisfaction. J Rheumatol. 2011;38:2461-5
