# Supplementary figures and images for: Relationships between psoriatic arthritis composite measures of disease activity with patient-reported outcomes in phase 3 studies of tofacitinib
Source: Arthritis Res Ther. 2021 Mar 26;23:94. doi: 10.1186/s13075-021-02474-2 (PMC7995583; doi:10.1186/s13075-021-02474-2)

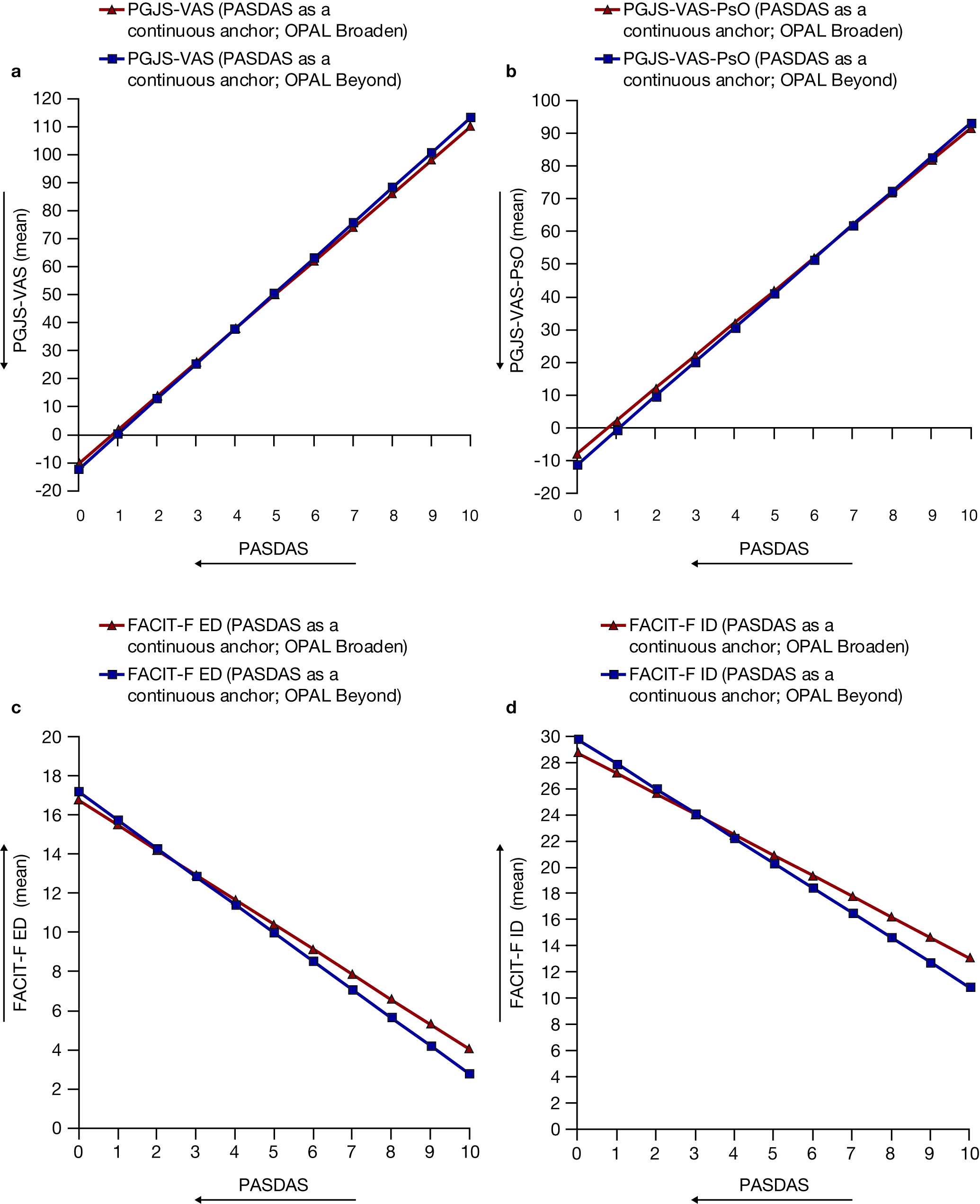

Supplement: Supplementary file 2 — Additional file 2 : Fig. S1. Estimated relationships between PASDAS and PGJS-VAS (a), PGJS-VAS-PsO (b), FACIT-F ED (c), and FACIT-F ID (d). Figure showing the estimated relationships between PASDAS as a continuous anchor and PGJS-VAS, PGJS-VAS-PsO, FACIT-F ED, and FACIT-F ID in OPAL Broaden and OPAL Beyond. [file 13075_2021_2474_MOESM2_ESM.tif]

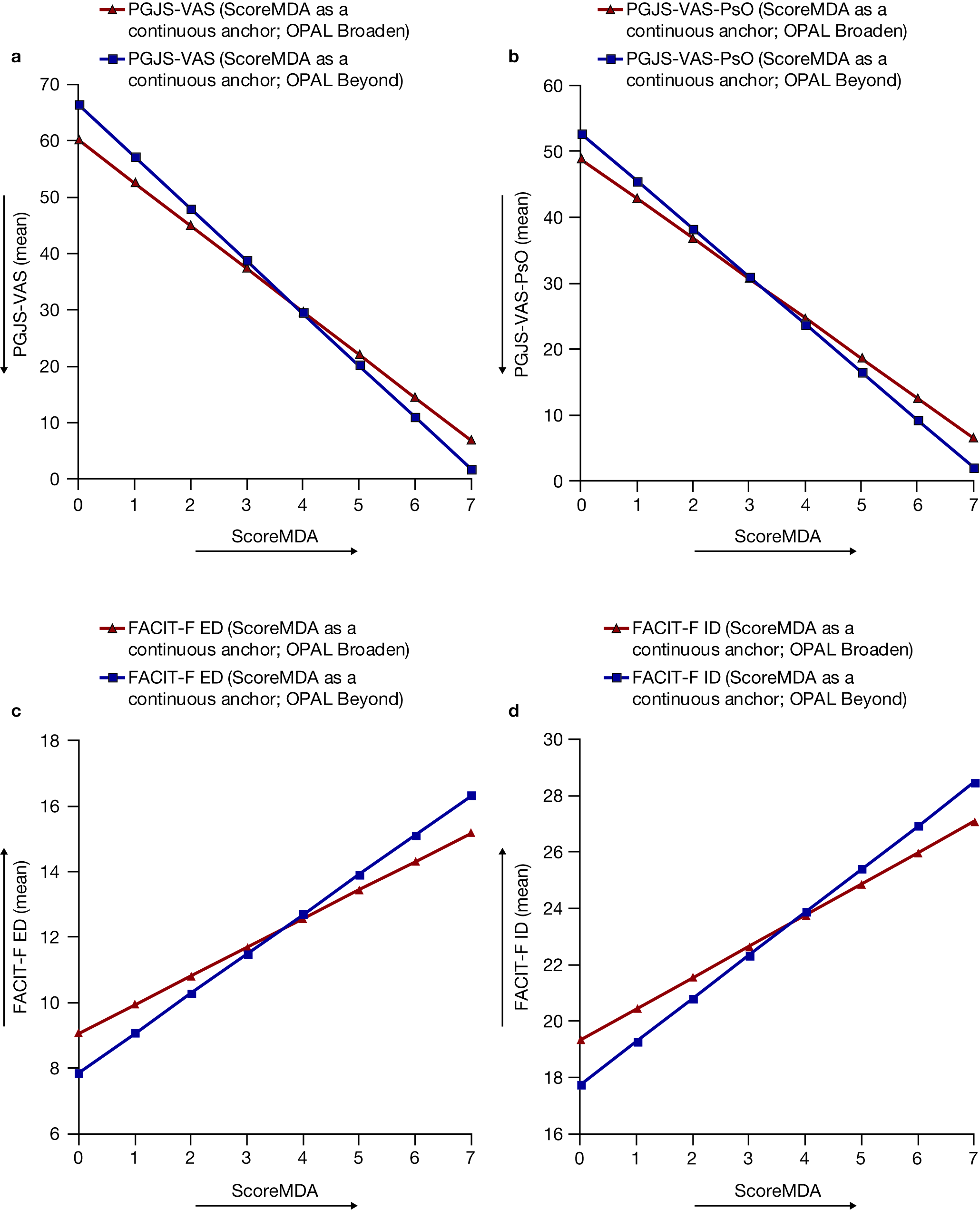

Supplement: Supplementary file 3 — Additional file 3 : Fig. S2. Estimated relationships between ScoreMDA and PGJS-VAS (a), PGJS-VAS-PsO (b), FACIT-F ED (c), and FACIT-F ID (d). Figure showing the estimated relationships between ScoreMDA as a continuous anchor and PGJS-VAS, PGJS-VAS-PsO, FACIT-F ED, and FACIT-F ID in OPAL Broaden and OPAL Beyond. [file 13075_2021_2474_MOESM3_ESM.tif]
